# The Asian brown cloud

**DOI:** 10.4103/0019-5278.43269

**Published:** 2008-08

**Authors:** Harshal T. Pandve

**Affiliations:** Department of Community Medicine, Dr. D. Y. Patil Medical College, Pimpri, Pune - 411 018, India

Dear Sir,

The most visible impact of air pollution is the haze, a layer of pollutants and particles from biomass burning and industrial emissions. This cloud of pollution at times has a brownish color (e.g., the Denver Brown Cloud) and this brown cloud phenomenon is a common feature of industrial and rural regions around the world. Due to long-range transport, the mostly urban (fossil fuel related) or rural (biomass burning related) phenomenon is transformed into a regional haze (or cloud) that can span an entire continent. It is now becoming clear that the brown cloud can have huge impacts on agriculture, health, climate and the water budget of the planet.[1] The haze consists of a combination of water droplets and minute particles. The water droplets in a haze are less than 0.001 mm in radius. There are two possible sources for the particles in a haze. They are either generated naturally (e.g., sea salt, dust) or man made (e.g., sulfate or soot). From an aircraft, the haze appears brown when the fraction of soot or dust is large. The Asian Brown Cloud is a layer of air pollution that covers parts of the northern Indian Ocean, India and Pakistan, and parts of South Asia, Southeast Asia and China.[2,3] This pollution layer was observed during the Indian Ocean Experiment (INODEX) intensive field observation in 1999. Subsequently, the United Nations Environment Programme (UNEP) has been supporting a project called ABC.[4] The potent haze lying over the entire Indian subcontinent - from Sri Lanka to Afghanistan - has led to some erratic weather, sparking floods in Bangladesh, Nepal and northeastern India but drought in Pakistan and northwestern India.[5]

The Asian Brown Cloud is rapidly melting Himalayan glaciers and could precipitate an environmental disaster that could affect billions of people. The effects have been linked to the retreat, over the last half a century, of glaciers in the Himalayas that supply water to major rivers including the Yangtze, the Ganges and the Indus. These rivers in turn comprise the chief water supply for billions of people in China, India and other South Asian countries. The consequences for China, India and other countries whose rivers flow from this source are incalculable, with the melting already being blamed for downstream flooding in late summer. Chiefly, domestic wood and dung fires plus smoke cause the cloud from the burning of forests and fields for agriculture. In addition, vehicle exhausts, power plants and factory chimneys add to the mix. However, the analysis of the pollution-filled clouds also offers hope that the region may be able to arrest the alarming retreat of such glaciers and ensure the security of water supplies by reducing pollution, for instance by cutting the dependence on wood-burning stoves.[6]

The INDOEX has revealed that this haze is transported far beyond the source region, particularly during December to April. The INDOEX findings pertain mainly to the period from December to April, referred to in this report as the dry season. This season is the ‘winter monsoon’ or the ‘north east monsoon.’ This UNEP report is the first comprehensive study of the South Asian haze and its impact on climate. It is largely based on the studies of the INDOEX science team of over 200 scientists from Europe, India and USA. It provides a summary of the large brownish haze layer and its impact on the radiative heating of the atmosphere and the surface for South Asia and the adjacent Indian Ocean during the INDOEX campaign. It also discusses preliminary findings with respect to the impact of this haze on regional temperatures, precipitation, agriculture and health.[7]

Haze can produce an impact on the agriculture productivity in a variety of direct and indirect ways. The direct effects are:

- Reduction of the total solar radiation (sum of direct and diffused) in the photosynthetically active part of the spectrum (0.4-0.7 µ) reduces photosynthesis, which in turn leads to a reduction in productivity.
- Settling of aerosol particles (e.g., fly ash, black carbon and dust) on the plants can shield the leaves from solar radiation.
- In addition, aerosol deposition can increase acidity and cause plant damage.

The indirect effects are:

- Changes in surface temperature can directly impact the growing season. In the tropics, a surface cooling (such as expected from aerosols) can extend the growing season (while a greenhouse warming can shrink it).
- Changes in rainfall or surface evaporation can have a large impact.[7]

There is now a growing body of literature linking air pollution with short-and long-term effects on human health. Populations at risk from inhaled particles are those most susceptible to pulmonary and heart diseases, infants and elderly people. A 1997 joint study of the World Health Organisation (WHO), the World Resources Institute and the US Environmental Protection Agency estimated that nearly 700 000 deaths worldwide are related to air pollution and that this number can escalate to 8 million deaths by 2020 (Working Group, 1997). Occurrences of respiratory diseases in South Asia resulting from air pollution, both indoors and outdoors, are estimated to be quite substantial. In each of the 23 cities with a population of over a million in India, air pollution levels exceed the WHO standards. It has been estimated that in India alone about 500 000 premature deaths are caused by indoor pollution in case of mothers and their children who are under 5 years of age. Serious respiratory disease - related problems have been identified for both indoor and outdoor pollution in Calcutta, Delhi, Lucknow, Bombay, Ahmedabad and several countries in East Asia, including China, Thailand and Korea. There is still inadequate knowledge about the relative effectiveness of submicrometer particles compared with larger particles or the specific roles of black carbon and organic carbon. Such studies need to be performed in the future.[7]

According to Srinivasan and Gadgil, The wide publicity given to the release of a UNEP report on the so-called Asian Brown Cloud and its multifarious impacts on health, agriculture and climate, on both regional and global scales, has led to considerable concern. The UNEP news release (and hence the media reports based on it) is a blend of observations and scientifically sound deductions on the one hand and sensational statements with little scientific basis on the other. The UNEP report is based on the findings of an international program called the INDOEX. The term Asian Brown Cloud was coined by leaders of the INDOEX to describe the brown haze occurring during the period of January to March over the South Asian region and the tropical Indian Ocean, Arabian Sea and Bay of Bengal. It is important to note that the haze is not a permanent feature of the atmosphere over the Asian region and the surrounding seas. It occurs only during January-March, in the season following the southwest monsoon and the northeast monsoon seasons. It is suggested in the UNEP report that the impact of the haze assessed with the help of an atmospheric general circulation model is a decrease in rainfall in northwest Asia (including Saudi Arabia, Pakistan and Afghanistan). However, the model simulation of the rainfall patterns over this region is particularly poor and hence the reliability of this report is suspicious. Also, the expected magnitude of the impact on crop yields is small and there is no basis for the statement in the UNEP news release that the ‘vast blanket of pollution across South Asia is damaging agriculture.’[2] Scientists in India are claiming that the Asian Brown cloud is not something specific to Asia and does not have a knock-on effect on pollution-related mortality. The environment ministry says that the report's drastic conclusions about disruption of weather patterns or massive monsoons, floods and draughts caused by the cloud were ‘unfounded’ as the report dealt only with the winter season over South Asia.[8]

According to Ramanathan *et al*., the South Asian brown haze covers most of the Arabian Sea, Bay of Bengal and the South Asian region. It occurs every year and extends from about November to April and possibly longer. The black carbon and other species in the haze reduce the average radiative heating of the ocean by as much as 10% and enhance the atmospheric solar radiative heating by 50-100%. These findings are in variance with Srinivasan and Gadgil's perceptions that the haze occurs only during January to March and that the aerosol forcing used by UNEP was unrealistically large because it used 1999 values and ignored the infrared (IR) effects of aerosols. The INDOEX and the UNEP did not rely solely on the 1999 values but used data from 1996 to 1999, and also accounted for the compensating IR effects. The long duration of the haze, its black carbon content, the large perturbation to the radiative energy budget of the region and its simulated impact on the rainfall distribution, if proved correct, have significant implications to the regional water budget, agriculture and health. The link between anthropogenic aerosols and reduction of monsoonal rainfall in South Asia has also been established using over 15 model studies preceding the UNEP report. The press release, while its direct quotes of the report are accurate, should have given more emphasis to the caveats in the report.[9]

To conclude, it is clear that the UNEP news release about the Asian Brown Cloud has created awareness about pollution. This should give an impetus to the ongoing program of reduction of harmful emissions in our cities. People living in Asia must be concerned about this haze because it has immediate and long-term impacts on their health.[2] The general population is always unaware about the recent environmental issues as a result of which the pollution is rising on a daily basis. This may be due to the increasing number of automobile vehicles, various industries or indoor pollution. Additionally, there is also a sudden rise in pollution during the festivals due to excessive fireworks. In India, so far work done related to global warming is mainly confined to research, conferences, seminars and workshops, with the general population having very little knowledge about the burning issue of global warming.[10] Similarly, there is little awareness about the Asian Brown Cloud in the general population. As global warming has emerged as matter of environmental concern for the entire world, similarly the Asian Brown Cloud is also one the essential environmental issues globally and especially for the Asian countries. All these growing environmental issues are somewhere interlinked either in causative factors or in the ways of preventing them. There is an urgent need to sensitize the general population about such growing environmental issues.
